# Multiple sclerosis susceptibility may be associated with the coding rs20541 (R130Q) *IL-13* gene polymorphism in the Polish population

**DOI:** 10.1038/s41598-023-49615-y

**Published:** 2023-12-12

**Authors:** Cezary Grunwald, Edyta Adamska-Patruno, Natalia Wawrusiewicz-Kurylonek, Agata Czarnowska, Katarzyna Snarska, Agnieszka Dardzińska-Głębocka, Katarzyna Kapica-Topczewska, Anna Mirończuk, Marcin Bazylewicz, Jan Kochanowicz, Adam Krętowski, Alina Kułakowska, Monika Chorąży

**Affiliations:** 1 Department of Neurology and Stroke Department, University Clinical Hospital in Bialystok, Białystok, Poland; 2 Clinical Research Support Centre, Medical University of Bialystok, Białystok, Poland; 3 Department of Nutriomics, Clinical Research Centre, Medical University of Bialystok, Białystok, Poland; 4 Department of Endocrinology, Diabetology and Internal Medicine, Medical University of Bialystok, Białystok, Poland; 5 Department of Clinical Genetics, Medical University of Bialystok, Białystok, Poland; 6 Department of Neurology, Medical University of Bialystok, Białystok, Poland; 7 Department of Clinical Medicine, Medical University of Bialystok, Białystok, Poland; 8 Department of Mechanics and Computer Science, Faculty of Mechanical Engineering, Bialystok University of Technology, Białystok, Poland

**Keywords:** Autoimmunity, Neurological disorders

## Abstract

Some of the multiple autoimmune diseases have been already associated with *IL-13* single-nucleotide polymorphisms (SNPs). However, there are only few studies regarding multiple sclerosis (MS) risk and *IL-13* rs20541 (R130Q) polymorphism, and their results are conflicting. Therefore, the aim of our study was to investigate the frequency of the *IL-13* gene rs20541 (R130Q) polymorphism in MS participants and its association with MS clinical subsets in the Polish population. We conducted a case‒control study including 94 relapsing remitting MS patients and 160 healthy volunteers. We genotyped the rs20541 polymorphism in the *IL-13* gene and analysed the genotype frequency, age of MS onset and clinical condition (EDSS values) of the MS participants. Fisher’s exact test was used for statistical analysis, and the log-linear model was applied to test for associations. Allele A, as well as the AA and AG genotypes, was observed to be significantly more common in the MS subjects. The OR (odds ratio) for the A compared to the G allele was 1.71 (1.14–2.56), whereas OR 2.33 (0.86–6.26) and OR 1.92 (1.11–3.30) were obtained for the AA and AG genotypes, respectively. We did not identify any significant associations of the studied *IL-13* SNP with the investigated clinical parameters of the MS participants. Our results suggest that the rs20541 polymorphism in the *IL-13* gene may play an important role in MS predisposition but not in investigated clinical parameters in MS subjects of the Polish population.

## Introduction

Multiple sclerosis (MS) is a chronic neurodegenerative disease involving autoimmune responses to myelin antigens associated with the activity of proinflammatory cytokines, which exert neurotoxic effects through various mechanisms (modulation of synaptic transmission and promotion of glutamate-mediated excitotoxicity, etc.)^1^. Neurodegenerative brain damage in MS is caused by an imbalance between proinflammatory cytokines released by T helper 1 (Th1) and T helper 17 (Th17) cells and anti-inflammatory cytokines released by T helper 2 (Th2) cells^2^. Interleukin 13 (IL-13), which is secreted mainly by Th2 lymphocytes, is one of the pleiotropic cytokines that may exert anti-inflammatory effects^3^. A study by Rossi et al.^4^ provided molecular, physiological and imaging evidence that IL-13 is efficiently involved in the modulation of neuronal integrity and synaptic function in individuals with multiple sclerosis, which suggests that treatments upregulating IL-13 production and release may have not only immunomodulatory effects but also neuroprotective benefits for these patients. Nevertheless, results related to the role of IL-13 in MS have been inconsistent among studies. Some findings suggest a pathogenic role for T-cell-derived IL-13 production in the central nervous system (CNS) in MS^5,6^.

Overall, MS is a chronic immune-mediated disease; however, it is also known that genetic predisposition contributes to its development. Genome-wide association studies (GWASs) have identified more than 200 genetic loci that show association with MS risk^7^, and molecular pathomechanisms may include effects on pre-mRNA splicing^8^. An impaired immune system response also includes a strong heritable component due to effects on the expression or biological activity of cytokines^9^. IL-13 is encoded by the *IL-13* gene located on the long arm of chromosome 5q31–33 in the cluster of genes encoding Th2 cytokines and contains four exons encoding a 146 amino acid protein^10^. Several single-nucleotide polymorphisms (SNPs) associated with immunological diseases have been identified in coding and noncoding regions of the *IL-13* gene^11^. Some of them (C-1112T, A-1512C) may influence the level of cytokine expression, whereas some (G+2044A) may have an impact on the affinity of IL-13 for its receptor^12,13^. Some SNPs (including rs20541 G+2044A (R130Q)) in the *IL-13* gene have been associated with chronic inflammatory and autoimmunological diseases, such as elderly associated chronic inflammatory diseases^14^, asthma^15^, autoimmune thyroid disease^16^, idiopathic nephrotic syndrome^17^, and food allergies^18^.

The association between the *IL-13* gene polymorphism rs20541 (R130Q) and MS has been investigated, to the best of our knowledge, by two other studies but only in the Iranian population^19,20^, which indicates that further investigations are needed, especially in different ethnicities. In the study by Mirahmadi et al.^20^ no relation between G+2044A (R130Q) and MS was observed, but associations of some *IL-13* gene SNPs were reported by Seyfizadeh et al.^19^. To our knowledge, there are no other studies in which the relationship between the rs20541 SNP and MS have been investigated. Overall, the studies to date suggest that ethnic differences cannot be excluded. In our study, we aimed to determine the frequency of the rs20541 (R130Q) polymorphism in MS and healthy control individuals in the Polish population and to analyse associations of genotype and allele with MS susceptibility and clinical disease manifestation.

## Results

The clinical characteristics of the MS study population are presented in Table 1. The frequencies of the investigated genotypes in the studied population are presented in Table 2. We did not observe any deviation from Hardy‒Weinberg equilibrium (chi-square test p value = 0.866). We noted that the AA and AG genotypes were more common in the MS population than in the healthy subjects (9.57% vs. 5.62% and 44.68% vs. 31.87%, respectively) but that the frequency of the GG genotype was higher in the control subjects than in the MS individuals (62.50% vs. 45.74%, respectively). These differences in genotype distributions were statistically significant, with a Fisher’s test p value of 0.031. Allele A was more common in the MS subjects (31.91% vs. 21.56%, p = 0.01), while allele G was more common in the control group (78.44% vs. 68.09%, Fisher’s test p value 0.011). When comparing the test statistic from Fisher’s exact test and the generalized linear model, the results were almost the same but not equal. The results of the generalized linear model, which uses the likelihood ratio statistic, confirmed the MS—rs20541 (R130Q) polymorphism association (p value 0.032). Genotypes AA and AG were significantly associated with an increased risk of MS compared to the GG genotype (OR 2.33 (0.86–6.26) and OR 1.92 (1.11–3.30), respectively). Compared to the G allele, the allele A was significantly associated with an increased risk of MS (OR 1.71 (1.14–2.56)).

**Table 1:** Clinical characteristics of the MS group.

Characteristic	MS subjects, n = 94	Women, n = 47	Men, n = 47
Age at onset (years)	41.15 ± 0.79	42.78 ± 0.98	37.14 ± 1.11
Disease duration (years)	8.12 ± 0.42	8.18 ± 0.51	7.96 ± 0.75
EDSS	1.91 ± 0.10	1.99 ± 0.19	1.83 ± 0.12
	1.5 (IQR 1–3)*	2 (IQR 1–3)*	1 (IQR 1–3)*

Data are presented as the mean ± SD.

*MS* multiple sclerosis, *EDSS* expanded disability status scale, *IQR* interquartile range.

*For EDSS, median values and IQRs are also presented.

**Table 2:** Genotypes and allele frequencies of the investigated rs20541 *IL-13* gene SNP in the studied groups.

Rs20541 genotypes/allele	MS (n = 94)		Control (n = 160)		P value	OR (95% CI)
	n	Frequency	n	Frequency		
AA	9	(9.57%)	9	(5.62%)		2.33 (0.86–6.26)
AG	42	(44.68%)	51	(31.87%)	0.03	1.92 (1.11–3.30)
GG	43	(45.74%)	100	(62.50%)		Ref
A	60	(31.91%)	69	(21.56%)		1.71 (1.14–2.56)
G	128	(68.09%)	251	(78.44%)	0.01	Ref

*SNP* single-nucleotide polymorphism, *MS* multiple sclerosis, *OR* odds ratio, *Ref* reference genotype/allele.

We did not identify any statistically significant differences in clinical data, such as age of onset or EDSS score, between the studied genotypes. The ages of MS onset for the AA, AG and GG genotype carriers were 45.0 (± 9.0), 43.6 (± 8.8) and 38.8 (± 10.0) years, respectively. Dunn’s test adjusted p values for the AA vs. AG, AA vs. GG, and AG vs. GG genotype comparisons were 0.60, 0.14 and 0.07, respectively. EDSS values for AA, AG and GG genotype carriers were 1.88 (± 1.09), 2.01 (± 1.29) and 1.93 (± 1.33), respectively, and we did not observe any statistically significant differences.

## Discussion

The rs20541 SNP (G+2044A, R130Q) of the *IL-13* gene has been associated with autoimmunological diseases. We sought to examine whether this variation in the *IL-13* gene may be associated with the susceptibility and clinical condition of individuals with MS of the Polish population. We noted significantly higher AA and AG genotypes, as well as A allele frequencies, in the individuals with MS, whereas the G allele and GG genotype were more frequent in the control group. Our analysis of ORs for the A allele and AA and AG genotypes suggests that the A allele may contribute to MS incidence in the studied population.

Our results are in contrast with results from a study by Mirahmadi et al.^20^, who did not observe any relation between G+2044A (R130Q) and MS. However, ethnic differences should be considered because a significant association for the investigated SNP polymorphism in patients of the Turkmen ethnic group of the Iranian population was noted. The association of some SNPs of the *IL-13* gene (including rs20541 (R130Q)) was observed in the Iranian population by Seyfizadeh et al.^19^.

The coding SNP rs20541 in exon 4 leads to a nonconservative guanine to adenine transition at nucleotide + 2044, leading to replacement of Arg 130 with Gln in α-helix D. This is a critical region and a significant amino acid for interactions between IL-13 and its receptors, and therefore, it may have an impact on IL-13-mediated signalling, resulting in increased biological activity and biological affinity between IL-13 and its receptor and may lead to inhibitory effects on immune responses^21^. However, the high frequency of the A allele in MS patients, as Seyfizadeh et al.^19^ pointed out, was not anticipated because the activity of IL-13 is decreased in MS patients. The protective role that is generally ascribed to Th2 cells has been questioned because a positive correlation has been observed between IL-13-producing cerebrospinal fluid (CSF) T cells and disability measured by the EDSS in RRMS patients^5^. Furthermore, a pathogenic phenotype with high production of IL-13 and other Th2 cytokines by brain-infiltrating T cells in demyelinating lesions has been identified^6^.

It is also worth emphasizing that IL-13 is involved in the maturation and differentiation of B cells, and an emerging role for B cells and the IgE pathway in triggering MS and other autoimmune diseases has been described^22^. In the study by Graves et al.^23^ carriers of rs20541 allele A had significantly higher total serum IgE levels than individuals carrying the G allele. A significant association between the rs20541 SNP and IgE serum concentrations was also noted in a study by Imraish et al.^24^, in which minor alleles were associated with a significant, approximately 5 times lower, total serum IgE level compared with the major alleles in individuals with asthma. All of these observations need further investigation with regard to the possible mechanisms to better understand the associations of *IL-13* gene polymorphisms with MS. We did not observe any association between the studied SNP and the age of MS onset or the clinical condition of MS subjects, and these results are in line with results from the study of Seyfizadeh et al.^19^, who did not observe associations between any investigated *IL-13* genotypes or alleles and the expanded disability status scale (EDSS) scores of Iranian participants.

When interpreting our results, the following limitations should be considered. The most important limitation is that we did not measure IL-13 or IgE concentrations in the study participants, which might provide more information about the observed associations. Nevertheless, this is the first pilot study in a Polish population investigating associations of MS with *IL-13* gene polymorphisms, and in future studies, more factors involved in the possible pathways should be included and analysed. Moreover, we focused only on the *IL-13* rs20541 variant, and it was already found that rs20541 is in strong linkage disequilibrium (LD) with, for example, another *IL-13* SNP, rs1295685, and that its haplotypes are associated with serum IgE levels^25^. Therefore, the possible impact of other SNPs being in strong LD or haplotypes should be considered in further analysis.

## Conclusions

In conclusion, our results suggest that the *IL-13* gene rs20541 polymorphism may play an important role in MS predisposition. We noted that allele A, as well as AA and AG genotypes, are significantly more common in MS participants of our study, with the ORs indicating the strength of the observed associations. However, investigated rs20541 SNP seems to be not associated with investigated clinical parameters, in MS subjects of the Polish population. Considering results from the other studies to date we can not exclude the impact of ethnic differences. An association observed in our study may be related to both mechanisms of SM development, T-cells and B-cells pathways as well, what we have discussed above. Nevertheless, further replication and functional studies are needed to investigate how common *IL-13* genetic variants may contribute to biological processes related to MS. More studies should also investigate other *IL-13* and *IL-13* receptor SNPs, as well as cytokine and other protein concentrations, to explore their clinical effects and roles in protecting individuals from or predisposing them towards MS development and progression.

## Methods

### Ethics

The study was approved by the Bioethical Committee of Medical University of Bialystok, Poland (R-I-002/334/2018). The study was conducted in accordance with the Helsinki Declaration, and all of the participants signed an informed consent form before enrolment in the study.

### Participants

The study group comprised 254 subjects: 94 with relapsing remitting MS (RRMS) (47 males and 47 females, mean age 41.15 ± 9.74 years, Table 1) and 160 unrelated healthy adults (85 males, 75 females; mean age 37.6 ± 2.16 years) without any family history of autoimmune diseases. The study population has been previously described in detail^26,27^, and it was recruited from the same ethnic group and from the same geographical area.

### Clinical disease manifestation

We analysed the age of MS onset and the clinical condition of MS subjects evaluated by Kurtzke’s Expanded Disability Status Scale (EDSS) at the time of diagnosis before treatment.

### Genetic analysis

DNA was isolated according to the manufacturer’s instructions with the Qiagen column separation method (QIAamp DNA Blood Mini Kit, Qiagen, Germany). We used a NanoDrop 2000 device (Thermo Fisher Scientific, USA) to evaluate the purity and concentration of the obtained preparations by a spectrophotometric method. All of the DNA samples were normalized to 50 ng/µl. The investigated SNP variant was tested in duplicate using the QuantStudio 12 K Flex platform on OpenArray plates (Thermo Fisher Scientific, USA) with TaqMan molecular probes applied to plates and Genotyping Master Mix (Thermo Fisher Scientific, USA). We analysed the rs20541 (R130Q, Gln144Arg) polymorphism in the *IL-13* gene. A sample without a template was used as a negative control to measure any false-positive signal caused by molecular contamination. The initial analysis of real-time PCR data was performed using TaqMan Genotyping Data Analysis Software—Genotyper (Thermo Fisher Scientific).

### Statistical analysis

Clinical parameters of the study participants measured as numerical data were characterized and are presented in tables by the number of observations, mean, and standard deviation (SD) or median and interquartile range (IQR). Categorical data such as allelic and genotypic characteristics are presented as the number of observations and frequency (%). Differences between numerical variables and the MS group were analysed using Dunn’s test. Allelic and genotypic frequencies were compared using Fisher’s exact test. The chi-square test was used to compare the observed genotype frequencies to the expected frequencies under Hardy‒Weinberg equilibrium (HWE). Moreover, we used a generalized log-linear model (glm) to test for associations. The significance level was set at < 0.05 for all 2-sided tests, and all calculations were performed using R version 4.1.2.

## Data Availability

The raw data supporting the conclusions of this article will be made available by the authors, without undue reservation, upon individual request from Dr hab. Monika Chorąży (chorazym@op.pl).
